# The Impact of Vitamin D on Dendritic Cell Function in Patients with Systemic Lupus Erythematosus

**DOI:** 10.1371/journal.pone.0009193

**Published:** 2010-02-16

**Authors:** Ilan Ben-Zvi, Cynthia Aranow, Meggan Mackay, Anfisa Stanevsky, Diane L. Kamen, L. Manuela Marinescu, Christopher E. Collins, Gary S. Gilkeson, Betty Diamond, John A. Hardin

**Affiliations:** 1 Division of Autoimmune and Musculoskeletal Disease, Feinstein Institute for Medical Research, Manhasset, New York, United States of America; 2 Department of Medicine, Albert Einstein College of Medicine, The Bronx, New York, United States of America; 3 Division of Rheumatology, Medical University of South Carolina, Charleston, South Carolina, United States of America; 4 Department of Medicine, Ralph H. Johnson Veterans Administration Medical Center, Charleston, South Carolina, United States of America; 5 National Institute of Allergy and Infectious Diseases, National Institutes of Health, Washington, D.C., United States of America; New York University, United States of America

## Abstract

**Background:**

Excessive activity of dendritic cells (DCs) is postulated as a central disease mechanism in Systemic Lupus Erythematosus (SLE). Vitamin D is known to reduce responsiveness of healthy donor DCs to the stimulatory effects of Type I IFN. As vitamin D deficiency is reportedly common in SLE, we hypothesized that vitamin D might play a regulatory role in the IFNα amplification loop in SLE. Our goals were to investigate the relationship between vitamin D levels and disease activity in SLE patients and to investigate the effects of vitamin D on DC activation and expression of IFNα-regulated genes in vitro.

**Methodology/Principal Findings:**

In this study, 25-OH vitamin D (25-D) levels were measured in 198 consecutively recruited SLE patients. Respectively, 29.3% and 11.8% of African American and Hispanic SLE patient had 25-D levels <10 ng/ml. The degree of vitamin D deficiency correlated inversely with disease activity; R = −.234, p = .002. In 19 SLE patients stratified by 25-D levels, there were no differences between circulating DC number and phenotype. Monocyte-derived DCs (MDDCs) of SLE patients were normally responsive to the regulatory effects of vitamin D in vitro as evidenced by decreased activation in response to LPS stimulation in the presence of 1,25-D. Additionally, vitamin D conditioning reduced expression of IFNα-regulated genes by healthy donor and SLE MDDCs in response to factors in activating SLE plasma.

**Conclusions/Significance:**

We report on severe 25-D deficiency in a substantial percentage of SLE patients tested and demonstrate an inverse correlation with disease activity. Our results suggest that vitamin D supplementation will contribute to restoring immune homeostasis in SLE patients through its inhibitory effects on DC maturation and activation. We are encouraged to support the importance of adequate vitamin D supplementation and the need for a clinical trial to assess whether vitamin D supplementation affects IFNα activity in vivo and, most importantly, improves clinical outcome.

## Introduction

Although patients with Systemic Lupus Erythematosus (SLE) exhibit diverse clinical and serologic manifestations, they share immunologic abnormalities resulting in loss of tolerance, autoreactivity, inflammatory sequelae and organ dysfunction. Non-toxic immunomodulatory modalities would be a welcome addition to the current agents used to manage autoimmune disease. Interferon alpha (IFNα) has multiple pro-inflammatory and permissive effects on autoreactive cells and has emerged as a central player in the progression to and maintenance of autoimmunity. It is produced in large amounts by activated plasmacytoid dendritic cells (pDCs). Therefore, therapies targeting IFNα and pDCs are currently in development for treatment of SLE. We and others are interested in the potential role of vitamin D deficiency in the development and perpetuation of SLE. In addition to its regulatory role in calcium balance and bone metabolism, vitamin D has immunomodulatory effects that help maintain immune homeostasis. We are particularly interested in the potential susceptibility of lupus pDCs to immunomodulation with vitamin D.

Vitamin D is a steroid hormone recognized as essential for bone and mineral homeostasis. Although small amounts of vitamin D are provided in the normal diet, the major portion is produced through the action of ultraviolet B radiation effects in the skin leading to the generation of cholecalciferol, the inert vitamin D precursor (vitamin D3). In the liver, a non-essential mitochondrial enzyme (CYP27A1) and an essential microsomal enzyme (CYP 2R1) convert Vitamin D3 to 25-hydroxy vitamin D (25-D). In renal tubule cells, 25-D is converted into the active 1,25-dihydroxy vitamin D (1,25-D) metabolite, calcitriol, by 1-alpha vitamin D hydroxylase (CYP27B1), Circulating levels of 1,25-D are controlled through complex mechanisms involving parathyroid hormone, calcium and phosphate concentrations, and self regulation which protect against vitamin D toxicity. Most of the biological effects of 1, 25-D are mediated through its interaction with the vitamin D receptor (VDR). In addition to cells in the gut and bone, the VDR is present in numerous cell types, including those of the immune system. It functions as an agonist-activated transcription factor that binds to vitamin D response elements in the promoters of many genes [1].

The immune system has all of the components needed to utilize vitamin D as an autocrine and paracrine signaling system. The VDR is expressed in T and B lymphocytes[2] and on monocytes differentiating into dendritic cells (DCs)[3]. Mature DCs express both CYP27A1 and CYP27B1and therefore are capable of locally generating the active 1, 25-D hormone from vitamin D3 [4]. In fact, *in vitro*, cultures of healthy donor DCs convert vitamin D3 into levels of 1, 25-D that are substantially higher than those typically measured in human serum providing further support that local generation of 1, 25-D at sites of an immune response may be a potential mechanism for modulating the immune system. Hormonally active vitamin D inhibits several components of the immune system[4], [5] including DC differentiation and maturation [6], B cell differentiation [7], T cell proliferation in response to T cell receptor (TCR) stimulation [2] and secretion of IL-12 and TNFα while IL-10 production is maintained[8]. Consistent with these findings, the hormone promotes Th-2 type T cell responses [9] and may promote generation of regulatory T cells [10].

A number of studies suggest that administration of vitamin D ameliorates inflammation in animal models of autoimmune diseases including lupus[11], [12], [13], multiple sclerosis[14], [15], and inflammatory bowel disease[16]. Vitamin D additionally inhibits graft rejection in multiple models of organ transplant (17). These animals studies are complemented by human epidemiologic studies demonstrating an inverse correlation between vitamin D intake with risk for multiple sclerosis[17], type 1 diabetes[18], and rheumatoid arthritis[19]. Although interventional studies using vitamin D as therapy for autoimmune disease are limited, short open label trials provide promising results in multiple sclerosis[20], psoriasis[21], and rheumatoid arthritis[22].

1, 25-D has suppressive effects on monocytes from healthy donors and inhibits IFNα-mediated monocyte differentiation into DCs. The effects of vitamin D on monocytes from autoimmune patients such as SLE are unknown. DCs comprise an integral link between innate and adaptive immune responses and can perpetuate an auto-inflammatory response. Immune complexes containing nucleic acids from apoptotic or necrotic cells trigger the production of IFNα. Following FcγR-mediated endocytosis and activation of Toll-like receptors (TLR) 7 and 9, pDCs are stimulated to release IFNα, that in turn promotes further differentiation of monocytes into DCs[23], [24]. Activated DCs can subsequently prime and polarize naïve T cells through expression of co-stimulatory molecules, and secretion of cytokines including IL-12, IL-10 or IL-23. Activated DCs will additionally promote activation and expansion of autoreactive T and B cells which sustain autoantibody production and continued release of IFNα[25], [26]. The central role of IFNα in SLE is clinically supported by observations that treatment with IFNα frequently results in autoimmune effects and, in some, a lupus-like disease. It is also clear that IFN inducible genes are over-expressed in peripheral blood mononuclear cells (PBMCs) in SLE patients [27], [28], [29], [30], [31]. This “IFN signature” correlates with disease activity and severity[32], suggesting that over production of this cytokine may be a driving force in SLE pathogenesis.

Vitamin D deficiency has been reported in patients with SLE[33] and it is known that 1, 25-D reduces healthy donor DC responses to Type I IFN maturation signals *in vitro* [6]. We hypothesized that vitamin D plays a regulatory role in the IFNα amplification loop in SLE patients. Our goals were to investigate the relationship between vitamin D levels and disease activity in SLE patients and to investigate whether vitamin D can modulate maturation and induction of the IFN signature in monocyte-derived DC (MDDC) from SLE patients. We describe a series of experiments that suggest the possibility that vitamin D supplementation will contribute to restoring immune homeostasis in individuals with SLE.

## Methods

### Participants

Consecutively encountered patients meeting ACR classification criteria for SLE were identified at three medical centers: the Medical University of South Carolina (Charleston, SC), the NIH clinical center (Bethesda, MD) and at the Montefiore Medical Center (Bronx, NY). Patients with a serum creatinine greater than 2.0 mg/dl were excluded. 198 subjects were recruited for analysis of 25-D levels and disease activity. Serum 25-D levels were assayed by the individual clinical center laboratories. Disease activity, measured using the Systemic Lupus Erythematosus Disease Activity Index (SLEDAI), patient demographics and medication use were also collected (Table 1). Prednisone dose was available for 144 subjects. Blood samples for the studies described below were collected from additional SLE patients and healthy controls at Columbia University Medical Center (New York, NY) and Jacobi Medical Center (Bronx, NY). All Phenotypic analysis of DCs: Heparanized blood (30 ml) was collected from 19 SLE patients with both stable disease activity and therapeutic regimens. Mononuclear cells were isolated over Ficoll-Hypaque and recovered cells were stained with flurochrome conjugated antibodies to CD11c, CD40, CD86, and HLA-DR and were analyzed by flow cytometry after gating on CD11c^+^ cells.

**Table 1:** Demographic and clinical characteristics of the patients.

		MMC and NIH groups			MUSC group
Race	Asians	African Americans	Caucasians	Hispanics	African Americans
Number of Patients (%)	6(6)	40(41)	10(10)	42(43)	100(100)
Age. Years (range)	41.8(19–53)	40.4(21–61)	48.7(17–69)	43.4(19–69)	39.1(10–70)
Female, no.	5	38	8	39	94
Male, no.	1	2	2	3	6
C3 mg/dl (range)	109(100–119)	100.5(27–162)	93.1(62–137)	97.14(38–166)	–
C4 mg/dl (range)	29(20–41)	23.6(7.8–48)	10(11–41)	22.44(7.8–68)	–
CH50 mg/dl (range)	–	–	–	–	25.7(0–59)
dsDNA, % positive	17	47	38	40	30
Serum creatinine mg/dl (range)	0.8(0.7–1.2)	0.9(0.5–1.8)	0.95(0.7–1.4)	0.9(0.5–1.8)	–
SLEDAI, median (range)	0(0–2)	2(0–22)	2(0–6)	4(0–10)	2(0–28)
Patients taking prednisone, no. (%)	6(100)	20(80)	7(70)	37(88)	–
Mean daily dose of prednisone in mg	5.2	18.2	8.5	15	–

Except where indicated otherwise, values are the mean (range). MMC  =  Montefiore Medical Center, NIH  =  National Institutes of Health.

MUSC  =  Medical University of South Carolina; SLEDAI  =  Systemic Lupus Erythematosus Disease Activity Index.

dsDNA  =  anti-double-stranded DNA.

### Generation of Monocyte Derived DC (MDDC)

Monocytes from SLE subjects and healthy donors were isolated from PBMC using RosetteSep Human Monocyte Enrichment Cocktail (StemCellTechnologies, Vancouver, Canada) according to the manufacturer's protocol. They were then cultured with 0.1 µg/ml IL-4 (Peprotech, Rocky Hill, NJ) and 0.27 µg/ml GM-CSF (Peprotech) for 5–7 days. Flow cytometry demonstrated a cell population comprised of 90% CD11c^+^ cells.

MDDC Activation Response: To study the effect of vitamin D on MDDC activation, MDDCs from 3 SLE patients were cultured with and without a 10 nM concentration of 1,25-D and stimulated with lipopolysacharide (LPS, Sigma) at a concentration of 50 ng/ml for 2 days. Surface activation markers, CD40, CD86 and HLA-DR were measured using flow cytometry.

### IFN Inducible Genes

1, 25-D-exposed or non-exposed MDDC from two healthy controls and two SLE subjects were incubated at 37°C for 4 hours with “activating plasma”, SLE plasma known to transfer the IFN signature, or healthy control plasma (20%) for 4 hours.. The incubation was performed in a 96 well flat bottom plate (Falcon, Franklin Lakes, NJ) with 5×10^5^ cells/100 µl /well in Eagles Minimum Essential Medium (EMEM) modified to contain Earl's balanced salt solution, non-essential amino acids, 2 mM L-glutamine, 1 mM sodium pyruvate and 1500 mg/l sodium bicarbonate supplemented with 10% FBS and 1% Penicillin G 10,000 units/ml and Streptomycin 10,000 units/ml. Following incubation with activating SLE plasma or healthy control plasma, RNA was extracted from the 1, 25-D exposed and non-exposed MDDC using Picopure RNA isolation kits (Arcturus Bioscience, Mountain. View, CA) followed by reverse transcription to generate complementary DNA (cDNA) in a 20 µl reaction using the iScript cDNA synthesis kit (BioRad, Hercules, CA). The cDNA was further subjected to quantitative PCR on a Bio-Rad Myiq cycler. Triplicates samples of cDNA were diluted 1∶10, and 7 µl was amplified in a 21 µl reaction mix using 2X TaqMan Universal PCR Master mix (Applied Biosystems, Foster City, CA) and 20X TaqMan Gene expression assay mix. Three IFNα-regulated genes were identified as target genes: Ifit1 (interferon induced protein with tetricopeptide repeats 1; Hs01675197 m1), Mx1 (myxovirus resistance 1; Hs00182073 m1) and Ifi44 (interferon induced protein 44; Hs00197427m1). These genes have been identified as highly and specifically induced by IFNα[34]. Expression of HPRT1 (hypoxanthine phosphoribosyltransferase 1) was monitored as the housekeeping gene. Relative expression of target genes was calculated using the comparative threshold cycle method.

### Ethics

The individual participants in this study gave written consent prior to any study procedures. Approval for the study was obtained from the National Institute of Health Sciences Institutional Review Board, the Committee on Clinical Investigations at the Albert Einstein College of Medicine and the Institutional Review Board for Human Research at the Medical University of South Carolina. As many of the translational studies were conducted at the Feinstein Institute for Medical Research, additional approval was obtained from the North Shore-Long Island Jewish IRB.

### Statistical Analysis

Correlations were determined by Pearson product moment correlation for interval data and by Spearman rank order correlation for ordinal data or for interval data, which did not follow a normal distribution. Nominal data were analyzed by chi-square analysis-of-contingency tables. Non-parametric Mann-Whitney tests were used for the between group comparison of surface markers in the DC phenotype.

## Results

### Vitamin D Deficiency Is Prevalent among Patients with SLE and Correlates with Increased Disease Activity

We obtained clinical and laboratory data on 198 consecutively encountered patients meeting ACR classification criteria for SLE. The majority were African Americans (71%) or Hispanics (21%) with relatively small numbers of Caucasians and Asians. The four racial groups had similar gender, age, renal function, SLE disease activity score (SLEDAI), serological findings and corticosteroid dose (Table 1).

**Figure 1:**
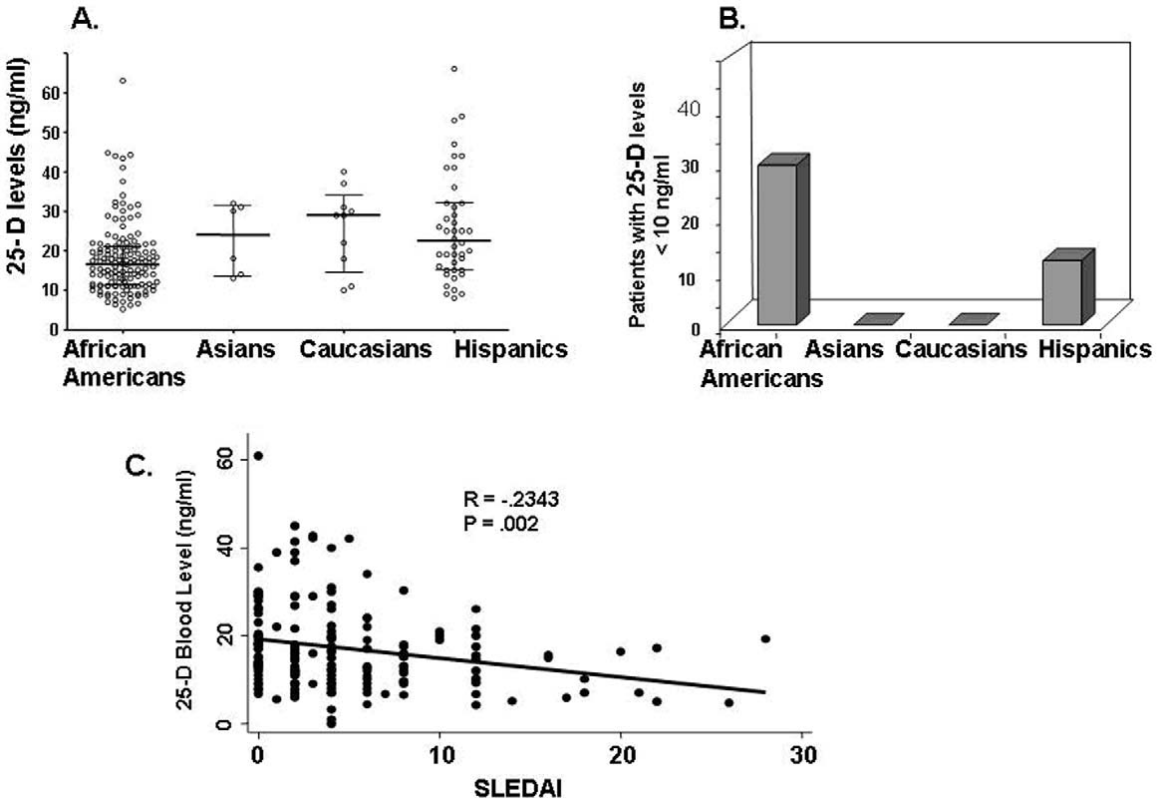
Assessment of 25-D levels in patients with SLE. The distribution and median blood level for 25-D (+/− 1 SD) is shown for each ethnic group (A). Severe vitamin D deficiency (defined as a blood level of 25-D less than 10 ng/ml) is most prevalent among African American and Hispanic patients (B). Disease activity scores (SLEDAI) exhibited an inverse correlation with 25-D levels (C).

Non-normalized median 25-D levels were lowest among African Americans (14.2 ng/ml, IQR 9.1–19) and Hispanics (20.5 ng/ml, IQR 13–30) and highest among Asians (22****ng/ml, IQR 11.5–29.5) and Caucasians (29 ng/ml, IQR 14.5–34) (Figure 1A). The prevalence of severe vitamin D deficiency among African Americans and Hispanics with SLE was striking; 29.3% and 11.8% respectively had 25-D levels less than 10 ng/ml. This level of deficiency was not observed among Asians and Caucasians (Figure 1B). Vitamin D levels correlated inversely with disease activity measured by SLEDAI (r = −.243, p = .002) even after controlling for ethnicity and prednisone dose (Figure 1C). There were no significant differences in 25-D blood levels between African Americans in South Carolina and those in the Bronx (data not shown).

### Circulating pDC and mDC Numbers and Phenotypes in SLE Are Not Affected by Vitamin D Levels

Previous studies of patients with SLE have demonstrated a fairly consistent decrease in circulating plasmacytoid DCs (pDCs) and a variable decrease in myeloid DCs (mDCs) compared to normal controls [35]. In addition, MDDCs from SLE patients are reported to be functionally different from normal DCs[36], [37]. Therefore, we initially sought to determine if vitamin D deficiency is associated with any major shifts in DC phenotypes. Peripheral blood mDCs and pDCs from 19 SLE patients were isolated by FACS and expression of activation markers on these circulating pDCs and mDCs were compared between groups stratified by 25-D levels of <20 ng/ml (n = 13) and >30 ng/ml (n = 6). All patients were on stable treatment regimens for SLE and both groups had comparable levels of disease activity. There were no significant differences in the numbers of circulating mDCs or pDCs between these two groups or any differences in expression of activation markers HLA-DR, CD-40, or CD-86 (data not shown).

### SLE MDDC Activation by LPS Is Modulated by 1,25-D

**Figure 2:**
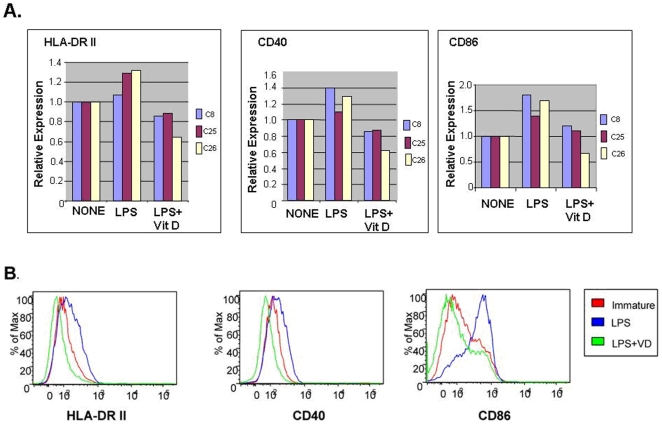
MDDCs from patients with lupus are responsive to the suppressive effects of vitamin D. DCs were prepared from monocytes of 3 African American patients with SLE (C8, C25, and C26) in the presence or absence of a 10 nM concentration of 1, 25-D and exposed to LPS as a maturation stimulus. Cells were analyzed for expression of HLA-DR II, CD40, and CD86 using flow cytometry. Composite results are shown as the relative value for mean fluorescent index compared to cells not exposed to vitamin D or LPS (A). The histogram data for one representative patient is shown (B). C8 was taking cellcept 3 gm/day and prednisone 5 mg/day, C25 was taking azathioprine 150 mg/day and prednisone 5 mg/day and C26 was taking methylprednisolone 8 mg/day. SLEDAI scores for C8 and C25 were 0 and the SLEDAI score for C26 was 4.

It has been previously reported that 1, 25-OH D3 added to healthy donor monocytes cultured *in vitro* with type I IFN and GM-CSF has a suppressive effect on MDDC maturation, differentiation and activation resulting in impaired LPS-induced production of Th-1 polarizing cytokines (5, 6). To determine whether the monocyte-DC axis in SLE patients is normally responsive to vitamin D, MDDC from 3 African American SLE subjects were prepared in the presence or absence of 10 nm 1, 25-D, followed by LPS stimulation. These patients had minimal disease activity (with SLEDAI ≤4), all were on low dose prednisone (≤10 mg/day) and two were on immunosuppressive medications (cellcept and azathioprine). Two of them had serum 25-D levels less than 30 ng/ml. Expression of activation markers HLA-DRII, CD40, and CD86 were measured by flow cytometry. Results are shown in Figure 2A; representative flow cytometry data for one subject is shown in Figure 2B. In the 3 lupus patients studied, MDDCs responded to LPS stimulation with increased expression of HLA-DRII, CD40, and CD86. This maturation response was blocked effectively by 1, 25-D as reported previously for DCs from non-autoimmune individuals[5]. Thus, MDDCs derived from SLE patients behave similarly to healthy donor MDDCs and are responsive to the constraints on maturation imposed by vitamin D.

### Vitamin D Inhibits Transfer of the IFN Signature to Healthy Donor and SLE MDDC In Vitro

**Figure 3:**
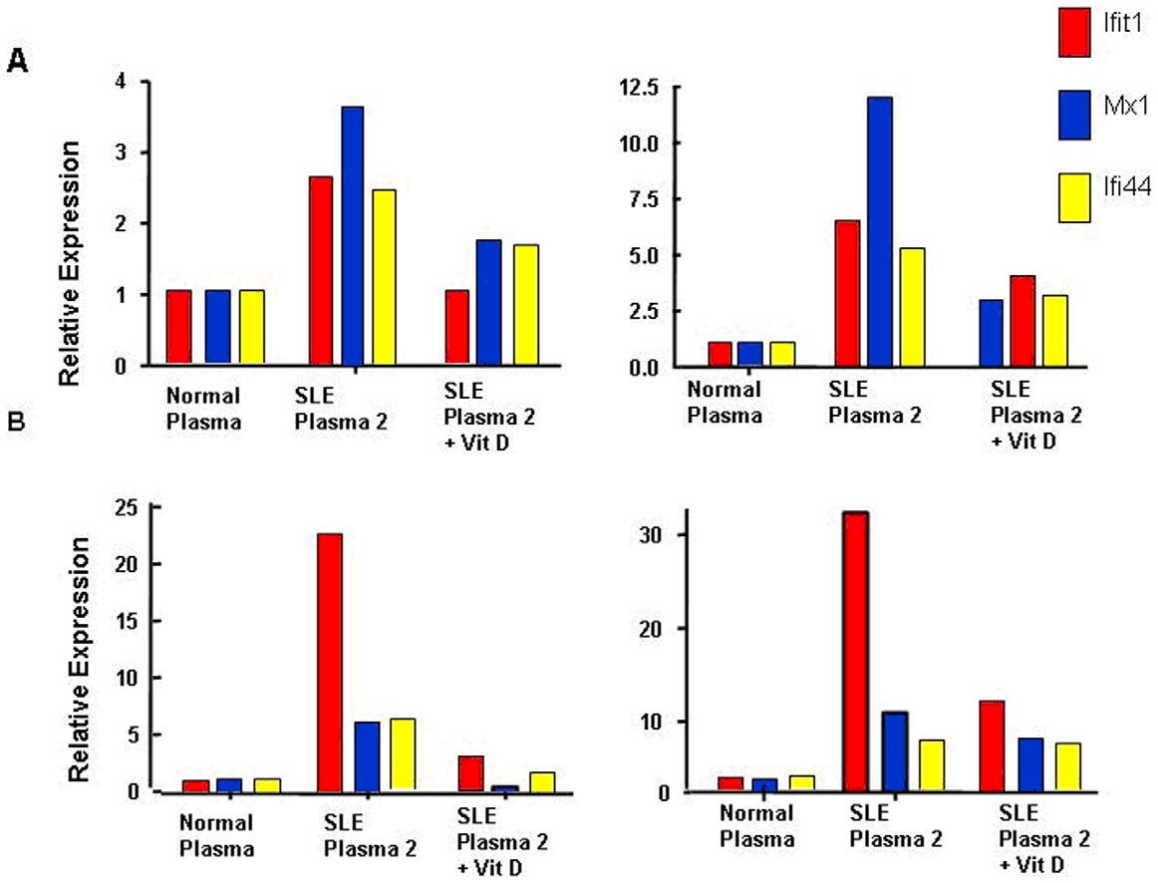
Vitamin D inhibits the induction of an IFN signature in MDDCs exposed to activating SLE plasma. Relative expression of three IFN inducible genes, compared to the housekeeping gene HPRT1, is shown in MDDC from 2 healthy donors cultured in the presence or absence of 1, 25-D at a concentration of 10 nM followed by exposure to activating SLE plasma or healthy donor plasma (Figure 3A). MDDC from 2 SLE subjects were subjected to an identical sequence of events (Figure 3B). One of these patients was an African American female with active renal disease, a SLEDAI of 10 and a serum 25-D level of 24. The other was a Hispanic female with SLEDAI score of 0 and a serum 25-D level of 19. Although expression of the 3 IFNα targeted genes appears to differ between healthy MDDC and SLE MDDC, overall expression is reduced with the addition of physiologic levels of Vitamin D during MDDC culture.

Type I interferons induce monocyte differentiation into DCs[6]. The ability of some lupus sera/plasma to induce DC differentiation has been attributed, at least in part, to the presence of IFNα and/or immune complexes within SLE sera that may trigger intracellular toll-like receptors(TLRs) and stimulate increased IFNα expression [38]. We assessed the impact of Vitamin D on the expression of IFN regulated genes (Mx1, Ifit1, Ifi44) induced by SLE activating plasma in healthy donor or SLE MDDCs. MDDCs from two healthy donors and two SLE subjects, exposed or non-exposed to 1, 25 D were incubated with activating SLE plasma or control plasma for 4 hours. One of these SLE patients had active renal disease (SLEDAI score 10), the other was clinically quiescent with a SLEDAI score of 0 and both had low serum levels of 25-D (24 and 19 ng/ml). As expected, healthy donor MDDC exhibited an increased expression of IFNα inducible genes when incubated with SLE activating plasma compared to healthy donor plasma (Figure 3A). This responsiveness to the stimulatory effects of SLE plasma was largely reversed in DC exposed to 1, 25-D (Figure 3A). Gene expression of MDDCs from both SLE subjects, one with active and one with inactive disease, was similarly responsive to stimulation with SLE activating plasma and modulation with 1, 25-D (Figure 3B). Overall percent reduction in gene expression modulated by the addition of 1, 25-D in healthy donor and SLE MDDCs was 62%, 60% and 34% for Ifit1, Mx1 and Ifi44 respectively, adding further evidence that MDDCs of SLE patients are responsive to the inhibitory effects of vitamin D.

## Discussion

It is well established that vitamin D is important for bone mineralization and homeostasis. Additional evidence is accumulating to suggest that this vitamin is important for many other aspects of health, including immune regulation. However, there are scarce data on the effects of vitamin D supplementation on the clinical status of patients with an autoimmune disorder. The present study provides support that vitamin D supplementation may help normalize immunologic abnormalities of SLE patients and offers insight into an immunomodulatory mechanism for vitamin D.

Our observations regarding vitamin D deficiency in SLE are consistent with previous reports of a high prevalence of vitamin D deficiency in SLE, particularly in African Americans [33], [39], [40], [41], [42], [43]. We found that 29% of African Americans with SLE from three centers had blood levels of 25-D less than 10 ng/ml compared to a previously reported prevalence of 12.1% in the same ethnic group in the NHANES III survey of healthy women of child bearing age [44]. Melanin is known to inversely correlate with endogenous production of vitamin D and lower serum levels of 25-D[45]. The striking prevalence of severely low vitamin D in African Americans with SLE may reflect additional non-identified disease related factors that further compromise body stores of vitamin D[45]. There was no discernable effect of sun exposure experienced by patients living at different latitudes on blood levels of 25-D in our patient cohorts although the study was not designed to assess this issue and lupus patients are routinely advised to avoid UV radiation.

Other studies have investigated the prevalence of vitamin D deficiency in SLE, and looked at a potential corrrelation of vitamin D levels with disease activity. Most, but not all, have reported an inverse correlation between disease activity and vitamin D levels [40], [46], [47], [48]. Our data confirm an inverse correlation between 25-D and disease activity. We have demonstrated that vitamin D deficiency may be permissive for increased disease activity in SLE because of its effects on the IFNα axis regulating DC differentiation and maturation. However, it is possible that the low vitamin D levels observed are reflective of an ongoing inflammatory process. Inflammation *per se* potentially enhances vitamin D catabolism[49] and anti-vitamin D antibodies have been reported in SLE patients[50]. SLE patients with more active disease are likely to receive higher corticosteroid doses. Our study did not show an association of corticosteroid use with serum vitamin 1, 25-D levels. It is possible that patients with greater disease activity spend less time in sun-exposed environments. Our data do not address this except to demonstrate no difference in serum 1, 25-D levels in patients in different latitudes.

Given the strong evidence for a fundamental role of IFNα in disease pathogenesis, we investigated the effects of vitamin D on DCs, specifically upon MDDC maturation and activation and on IFNα activity *in vitro*. Prior to investigating the potential effects of IFNα on MDDCs, we established that circulating DC numbers and phenotype *in vivo* were not affected by serum levels of vitamin D. Two groups of SLE subjects stratified by 25-D levels with comparable medication doses and disease activity had equivocal numbers and surface expression markers of mDCs and pDCs. The focus of the remainder of our studies was on the effects of vitamin D on MDDCs. We report that lupus MDDCS behave similarly to normal MDDCs and are responsive to the effects of vitamin D *in vitro.* Vitamin D exposure promotes an immature, tolerogenic DC phenotype. Vitamin D additionally inhibits the overexpression of INF inducible genes. These investigations are clearly limited by the small numbers of samples studied. However, the consistency of the data, from both lupus and normal controls strongly suggests that our analyses are not incidental findings.

IFNα is a pleiotropic Type I interferon that has been implicated in SLE disease pathogenesis. As previously discussed, treatment of malignancy and chronic hepatitis with recombinant IFNα resulted in increased autoantibody production and, occasionally, clinical features suggestive of SLE that resolve following cessation of treatment[51]. It is also known that IFNα has the ability to regulate DC differentiation after phagocytosis of apoptotic self-debris leading to a break in tolerance[38]. Nucleic acid containing immune complexes are also able to stimulate DCs through TLR activation and result in increased IFNα production. IFNα then drives differentiation and maturation of mDCs in an autocrine fashion, resulting in stimulation of autoreactive T and B cells and further immune complex formation[52]. Many SLE patients have evidence of increased IFNα activity demonstrated by the overexpression of IFNα regulated genes (the IFN signature)[28] or increased serum IFNα levels. Familial aggregation of high IFNα serum levels as well as the presence of the IFNα signature among SLE patients and families suggests heritable risk factors related to IFNα[53]. Several recently identified SLE susceptibility genes including IRF5, STAT4 and PTPN22 are associated with increased serum IFNα activity that is related to IFNα production and downstream sensitivity to IFNα[54], [55], [56].

Vitamin D potentially regulates MDDCs by several mechanisms including effects on the nuclear factor-*k*B (NF-*k*B) transcription factor, RelB. The RelB promoter region contains a VDR binding site; ligation of the VDR at this sites reduced transcription of RelB[57]. RelB is an essential element for DC differentiation and maturation and is known to regulate MDDC subset development [58], [59], [60]. Vitamin D additionally augments cellular levels of I*k*Bα, potentially reducing cellular sensitivity to ligands that depend upon NF-*k*B signaling pathways[61]. In addition, vitamin D modulates expression of TLRs whose signals synergize with IFNα responses[62] and down regulate the type 1 interleukin-1 receptor [63] which serves a stimulatory role in the TLR cascade[64]. Finally, vitamin D reduces expression of many genes that are important for the amplification of the immune response including IL-2, IFNα, and CD40.

It is certainly appealing to consider that the mechanism by which vitamin D repletion might benefit patients with autoimmunity is based on the ability of DCs and other cells of the immune system to locally produce hormonally active 1, 25-D from the inert substrates cholecalciferol and 25-D. Through up-regulation of 1α-OHase, DCs generate concentrations of 1, 25-D in culture that approximate the pharmacological concentrations used for suppression of MDDC maturation and inflammatory cytokine secretion[65], [66] Increased local DC production of 1, 25-D has been proposed as a potential mechanism for controlling autoimmune responses in inflammatory sites.

These studies demonstrate that individuals with SLE have MDDCs that display normal responsiveness to vitamin D resulting in decreased activation. These studies additionally show that vitamin D inhibits the overexpression of IFN regulated genes in both normal and SLE derived MDDCs that are exposed to activating plasma. It is unclear if vitamin D insufficiency confers an increased likelihood of a genetically susceptible individual to progress to a full autoimmune disease or if vitamin D insufficiency is permissive for increased immunologic activation and disease activity or both. Kamen et al, examining a different patient cohort [47], reported lower serum 25-D levels among recently diagnosed SLE patients suggesting that vitamin D deficiency may be a risk factor for the development of SLE. Our current study and others show an inverse relationship between vitamin D levels and disease activity in established SLE. It is therefore possible to ask whether increased serum vitamin D levels might lead to immunologic and subsequent clinical improvement and so to demonstrate conclusively whether vitamin D levels contribute to disease activity. However, it is difficult to predict the amount of vitamin D supplementation required to restore 25-D levels to a desired range. New norms for vitamin D have been suggested through meta-analyses relating serum levels of 25-D to fractures, falls and neoplastic disease. These studies indicate that optimal levels of 25-D should be above 30 ng/ml, however, it is unknown whether the desired level for immune homeostasis is the same or different of that needed for optimal bone health. Abrogation of the IFN signature in SLE PBMC provides a measurable response to vitamin D supplementation. The results of an ongoing phase I trial studying the safety of 3 doses of vitamin D in SLE (ClinicalTrials.gov Identifier: NCT00710021) and the results of a phase II trial studying the effects of vitamin D supplementation on the IFN signature are eagerly awaited.

In summary, vitamin D supplementation potentially provides a non-toxic approach to improving immune regulation in SLE patients. Our study contributes information on the prevalence of vitamin D deficiency in SLE and association with disease activity. It is the first study to demonstrate the inhibitory effects of 1, 25-D on SLE MDDC activation and transfer of the IFN signature *in vitro* and to show that MDDCs derived from SLE patients are responsive to changes in 1, 25-D concentration. The study is limited by the small samples sizes used in the experiments. Adequately powered studies are needed to determine how best to replenish body stores of vitamin D in patients with SLE and to determine whether such supplementation is associated with a reduction in IFNα activity and disease activity in patients with this disease.
